# SNP discovery and molecular evolution in *Anopheles gambiae*, with special emphasis on innate immune system

**DOI:** 10.1186/1471-2164-9-227

**Published:** 2008-05-19

**Authors:** Anna Cohuet, Sujatha Krishnakumar, Frédéric Simard, Isabelle Morlais, Anastasios Koutsos, Didier Fontenille, Michael Mindrinos, Fotis C Kafatos

**Affiliations:** 1 Institut de Recherche pour le Développement, UR 016, BP 64501, 911 Avenue Agropolis, 34394 Montpellier Cedex 5, France; 2 Stanford Genome Technology Center, 855 California Avenue, Palo Alto CA 94304, USA; 3 Organisation de Coordination pour la lutte contre les Endémies en Afrique Centrale, Laboratoire de Recherche sur le Paludisme, BP 288, Yaounde, Cameroon; 4 Imperial College London, Division of Cell and Molecular Biology, Sir Alexander Fleming Building, South Kensington Campus, London, SW7 2AZ, UK; 5 BMC-series Journals, BioMed Central, Middlesex House, 34-42 Cleveland Street, London W1T 4LB, UK

## Abstract

**Background:**

*Anopheles *innate immunity affects *Plasmodium *development and is a potential target of innovative malaria control strategies. The extent and distribution of nucleotide diversity in immunity genes might provide insights into the evolutionary forces that condition pathogen-vector interactions. The discovery of polymorphisms is an essential step towards association studies of susceptibility to infection.

**Results:**

We sequenced coding fragments of 72 immune related genes in natural populations of *Anopheles gambiae *and of 37 randomly chosen genes to provide a background measure of genetic diversity across the genome. Mean nucleotide diversity (π) was 0.0092 in the *A. gambiae *S form, 0.0076 in the M form and 0.0064 in *A. arabiensis*. Within each species, no statistically significant differences in mean nucleotide diversity were detected between immune related and non immune related genes. Strong purifying selection was detected in genes of both categories, presumably reflecting strong functional constraints.

**Conclusion:**

Our results suggest similar patterns and rates of molecular evolution in immune and non-immune genes in *A. gambiae*. The 3,214 Single Nucleotide Polymorphisms (SNPs) that we identified are the first large set of *Anopheles *SNPs from fresh, field-collected material and are relevant markers for future phenotype-association studies.

## Background

*Anopheles gambiae*, the main vector of the human malaria parasite *Plasmodium falciparum *in SubSaharan Africa, is the most medically relevant insect in the world. Together these two organisms are responsible annually for more than a million of deaths in Africa, mostly young children. This epidemic is worsening [1], prompting the search for innovative strategies towards effective and efficient malaria control. One approach aims to disrupt parasite development in the mosquito vector and thus alleviate malaria transmission intensity [2]. This strategy requires clear understanding of the intimate interactions between parasite and vector, and of the mechanisms that regulate the interaction. Large scale gene expression profiling in *A. gambiae *has revealed that the insect's innate immune system is stimulated following infection by *Plasmodium *parasites [3-6], highlighting this system as a primary candidate for interventions to control the infection. Extensive studies identified potentially relevant mechanisms of innate immune response, including a balance between positive and negative mosquito factors towards the parasite [7-10]. Genetic variation underlies the susceptibility of *A. gambiae *to *Plasmodium *infection: refractory mosquito strains have been selected [11,12] and QTLs identified for susceptibility/refractoriness to the model parasite, *P. cynomolgi *[13,14]. However, to date the mechanism(s) involved and the underlying genetic basis remain unknown [15]. Some studies conducted in field conditions with wild mosquito and parasite populations further demonstrated genetic variability in the mosquito's susceptibility to *P. falciparum *infection and identified putatively involved genes [16-18]; their contribution to the phenotype and relevance *in natura *still remain to be assessed fully.

The availability of the *A. gambiae *genome sequence [19] has opened new perspectives for exploratory genetic studies in this species. The activities and evolution of its immune system are now being studied intensively, and could provide insights into the past and present patterns of interaction with the pathogen. Initial studies revealed selective constraints of diverse nature acting on some immunity genes [20-24]. The current pattern of malaria transmission is relatively recent (less than 10,000 years), but has exerted strong selective pressure on human populations that led to the selection of resistance alleles, some of which are strongly deleterious [reviewed in [25]]. Signatures of selection were found on most genes implicated in *P. falciparum *resistance in humans [26] and conversely, population studies on *P. falciparum *have detected selection sweeps on its genome [27]. It is rational to expect that *P. falciparum *has also exerted selective pressure on *A. gambiae*. If so, evolutionary genetics of the immune system in natural *A. gambiae *populations should pinpoint resistance or susceptibility genes based on their peculiar molecular makeup.

Single nucleotide polymorphisms (SNPs) are the commonest mode of genetic variation in vertebrates and invertebrates [28-30]. As such, SNPs rapidly became the preferred and most useful molecular markers for association studies, high resolution linkage mapping and population genetics studies [26,31,32]. In coding regions, synonymous SNPs (sSNPs) that do not result in amino-acid change are likely neutral markers for population genetic studies. In contrast, non synonymous SNPs (nsSNPs) alter protein structure and could be retained by natural selection. The search for SNPs in *A. gambiae *immunity genes is an initial step towards genetic dissection of vector competence in the wild [21].

Here, we report coding region SNPs in a representative set of genes from different families associated with putative innate immune functions in *A. gambiae *[5]. Genes chosen randomly across the genome were included for comparison with the immunity data, such that the genome-wide effects of demographic history may be distinguished from gene-specific effects of selection. The study was carried out on natural populations of *A. gambiae s.s*. (both M and S molecular forms) and its sibling species, *A. arabiensis*, collected from field sites in Cameroon (Central Africa).

## Results

**Figure 1:**
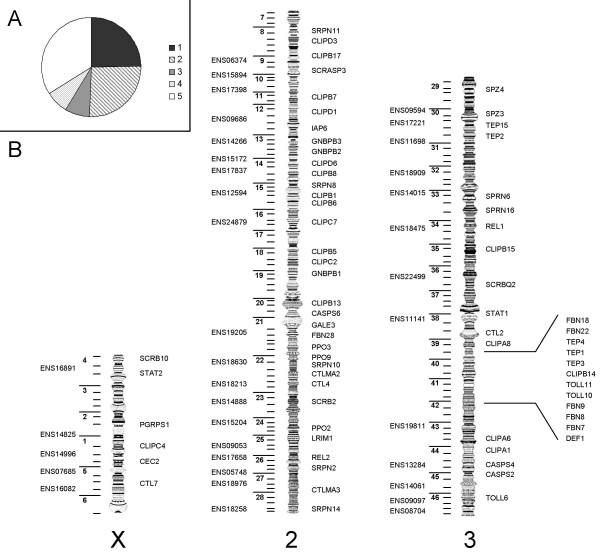
**Studied genes**. A: Proportion of studied genes putatively involved in (1) Recognition, (2) Modulation, (3) Signal transduction and (4) Effectors molecules of immune response and (5) Non imnnune related genes. B: Relative position of studied genes on the *Anopheles gambiae *genome. Immune related genes are indicated at the right of each chromosome, non immune related genes on the left by using the ENSEMBL ID reduced to the three first letters and five last numbers.

We studied 72 immune related genes, representing innate immunity gene families and functions throughout the genome [5]. Additionally, 37 non-immune related genes randomly chosen along the genome were included in the study. The relative proportion of genes involved in the different stages of immune response [5] and their chromosomal location are shown in Figure 1. Target genes are listed in tables [see Additional files 1 and 2], with their Ensembl gene IDs, accession numbers in the EMBL database, their putative role in the immune response (functional class), primer sequences, gDNA or cDNA nature of the template, chromosomal location, length of the fragment analyzed, and the number of alleles sequenced.

### Sequence Polymorphism

**Figure 2:**
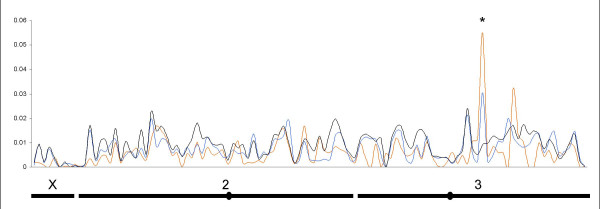
**Nucleotide diversity**. Nucleotide diversity along the chromosomes for *A. arabiensis *(red line), *A. gambiae *M form (blue line) and *A. gambiae *S form (black line). Data from immune related genes and control genes are included. Abscise represents position of the genes along the genome. Chromosomes and centromeres are represented below. The asterisk shows the position of TEP1 gene.

We analyzed a total of 2,608,472 nucleotides across 109 coding fragments of an average length of 524 base pairs. Nucleotide diversity indices and results of the tests for selection within populations are given in Additional files 3 and 4. A total of 3,214 SNPs were detected in our dataset: 2,026 were observed in immune related genes and 1,188 in non immune related genes, respectively. Correspondingly, 1,711 and 1,071 SNPs were newly identified polymorphisms (not previously reported). We also detected 54 indels, always as a multiple of 3 bases preserving the open reading frame (ORFs). In the populations we studied, 432 (78%) of the 554 SNPs previously reported in ENSEMBL were detected (data not shown). Nucleotide diversity along the chromosomes is presented Figure 2, including immune related genes and control genes. Mean nucleotide diversity (π) across all genes varied significantly between species and populations (Mann-Withney U test, P < 0.05): it was higher in the *A. gambiae *S form (π = 0.0092) than in either the M form (π = 0.0076) or in *A. arabiensis *(π = 0.0064). Within each species, however, no statistically significant differences in mean nucleotide diversity was detected between immune related and non immune related genes, nor between different functional class within immune related genes (P > 0.05). Similarly, nucleotide diversity was evenly spread on the four autosomal arms in the three populations. However, even after correction for lower effective population size on the X chromosome by multiplying estimates by 4/3 (see Methods), significantly lower genetic diversity (P < 10^-3^) was observed on the X chromosome in each population (mean π: 0.00131 for *A. arabiensis*, 0.00319 for the M form and 0.00358 for the S form). Noticeably however, the gene TEP1 on the third chromosome showed much higher genetic diversity than all the other genes we investigated (Figure 2).

### Species divergence and population differentiation

**Figure 3:**
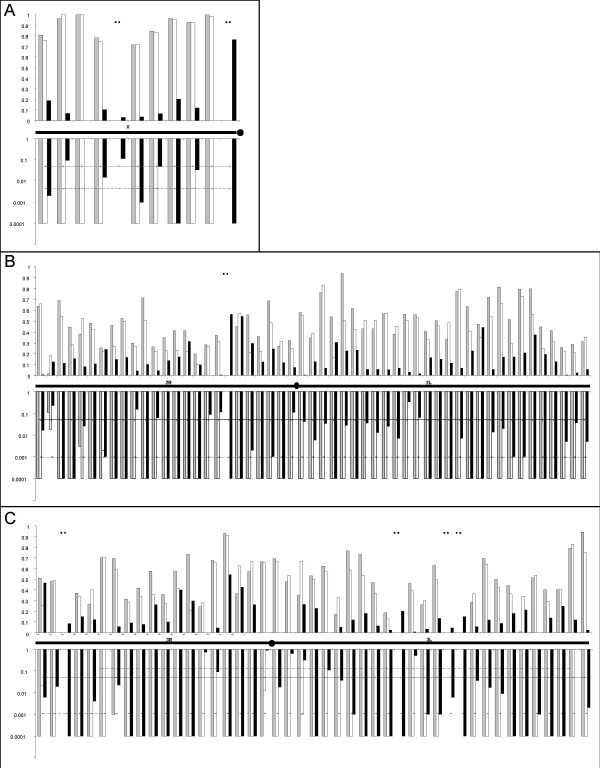
**Genetic differentiation between populations**. Genetic distance between pairs of populations on chromosomes X (A), 2 (B) and 3 (C). Fst estimates are shown in the top part of each graph and the corresponding P-values are shown below, in grey for *A. arabiensis*/*A. gambiae *M form comparisons, white for *A. arabiensis*/*A. gambiae *S form and in black for *A. gambiae *M form/*A. gambiae *S form. Data from immune related genes and control genes are included. Horizontal straight lines represent the significance threshold at P= 0.05, dashed lines: P = 0.05 after correction for multiple tests (Bonferroni sequential procedure). Missing data are indicated with a dot. Negative Fst values that are always not significant were represented as equal to zero. Highly significant P values (<10^-4^) were represented as P = 0.0001 to be shown on a logarithmic scale.

Pairwise estimates of genetic differentiation (Fst) between all population pairs are given on Figure 3, together with their statistical significance, for each of the 109 genes. Similar levels of genetic differentiation were detected by immune related and non immune related genes, as well as between different functional classes within immune related genes (Mann-Whitney U test on Fst estimates, P > 0.05). Hence, data from all categories of genes were pooled for further analyses. Average Fst estimates between the M and S molecular forms of *A. gambiae *was similar across all 3 chromosomes (Mann-Whitney U test on Fst estimates, P > 0.05), with an overall Fst = 0.1377 (P < 10^-3^). Allelic frequencies differed among the incipient species but only one sSNP found in a non-immune related gene in section 5D on the X chromosome **(**ENSANGG00000016082) segregated between the M and S forms. Mean genetic divergence between *A. arabiensis *and either the M or S form were similar (Fst = 0.5268 and Fst = 0.4729 respectively, Mann-Whitney U test P > 0.05), and were significantly higher than between the two molecular forms (P < 10^-3^). Between *A. arabiensis *and the M or S form, 175 and 125 SNPs, respectively were fixed and mean genetic differentiation estimates were significantly higher on the X chromosome (P < 0.01).

### Tests of selection in immune related and non-immune related genes

The Tajima D statistic was computed for each gene in all populations [see Additional files 3 and 4]. At equilibrium between random genetic drift and mutation, the expected value of D for neutral markers is close to zero. This statistic detected only few genes with significant departure from neutrality, reflecting locus-specific selection rather than the effect of demographic instability. Noteworthy, TEP1 showed a highly significant positive value of D in *A. arabiensis *(P < 0.01). This departure from neutrality was due to the coexistence, at high frequency in this population, of two highly diverged alleles: the previously described and widespread TEP1r and TEP1s [7] were observed at a relative frequency of 37.5% and 62.5%, respectively, in *A. arabiensis*. In *A. gambiae*, TEP1s was the most frequent allele and TEP1r was found at low frequency (12.5% in the M form).

The Z test of selection revealed a very high proportion of genes that are under selection across populations: only 3 genes among the immune related genes (3/72 = 4.1%) and one among the non immune related genes (1/37 = 2.7%) showed the ratio of sSNPs/nsSNPs expected under the hypothesis of neutrality. All other genes are strongly deficient in non-synonymous changes, suggesting prevalence of purifying selection. At the population level, the test was less often significant, most likely reflecting the lack of statistical power when sequences are not sufficiently polymorphic [33].

For each pair of populations, Ka/Ks ratios are shown in Additional files 5 and 6 for immune and non immune related genes, respectively. Ka/Ks ratios are expected to equal 1 if the genes under scrutiny behave neutrally. In most case, however, and for both immune and non immune related genes, the Ka/Ks ratios were much lower than 1. Such a pattern implies that a mutation changing the encoded amino acid sequence is much less likely to be different between two species than one which is silent. This is consistent with the results of the Z test of selection and suggests that purifying selection is a major force driving the evolution of both immune and non immune related genes in all populations.

Lack of fixed mutations precluded the implementation of the McDonald-Kreitman test between *A. gambiae *molecular forms. For the comparison of *A. arabiensis *with the two *A. gambiae *molecular forms, the test was computed across groups of genes in order to encompass a sufficient number of fixed variations for building the contingency tables. However, combining data from different genes for MacDonald-Kreitman tests has established drawbacks which can lead to spurious cases of positive selection [34], thus significant tests need to be carefully interpreted. Table 1 shows the total numbers of replacement and silent polymorphisms that are shared or fixed between pairs of species, across immunity and non immunity related genes. For the latter group, the test revealed adequacy with neutral expectations (Two tailed Fisher exact test: P = 0.426 and P = 0.622 for each pair of species, respectively). Deviations from neutrality in immune related genes were at the limit of the significance threshold for both species pairs (Fisher exact test: P = 0.038 and P = 0.081, respectively) leading to inconclusive results as a reflection of limited statistical power in our dataset.

**Table 1:** McDonald and Kreitman test results in immune and non immune related genes

A: Between *A. arabiensis *and M form for immune related genes			
	Non synonymous	Synonymous	Ratios

Interspecific fixed divergences	36	63	0.571
Intraspecific polymorphisms	344	905	0.380
Ratios	0.104	0.070	P = 0.081

B: Between *A. arabiensis *and S form for immune related genes			

	Non synonymous	Synonymous	Ratios
Interspecific fixed divergences	24	37	0.649
Intraspecific polymorphisms	421	1171	0.360
Ratios	0.057	0.032	P = 0.038

C: Between *A. arabiensis *and M form for non immune related genes			

	Non synonymous	Synonymous	Ratios
Interspecific fixed divergences	16	55	0.290
Intraspecific polymorphisms	151	675	0.224
Ratios	0.106	0.081	P = 0.426

D: Between *A. arabiensis *and S form for non immune related genes			

	Non synonymous	Synonymous	Ratios
Interspecific fixed divergences	14	49	0.286
Intraspecific polymorphisms	192	798	0.241
Ratios	0.073	0.061	P = 0.622

SNPs were counted for the groups of genes (immune related or non immune related genes) within species (Intraspecific polymorphisms) or between species (Interspecific fixed divergences). The probability (P) is calculated using a 2-tailed Fisher's exact test.

## Discussion

This study provides the most extensive set of data on molecular polymorphisms in immune and non immune related genes in *A. gambiae *and *A. arabiensis*. We observed a high level of nucleotide diversity (π>0.006) in coding regions within members of the *A. gambiae *complex. This is approximately ten fold higher than the level of nucleotide diversity observed in coding regions of the human genome [29] but is comparable to the estimates reported from *Drosophila *or in previous studies on *Anopheles *[20,21,24,35-38]. Nucleotide diversity is a product of both mutation rate and effective population size. Mutation rates were shown to be similar in humans and *Drosophila *[39,40], so the ten fold difference in average nucleotide diversity between species was attributed to the approximately ten fold larger long term effective population size in *Drosophila *than in humans (Ne = 300,000 versus 20,000 respectively) [41,42]. The effective population size of *A. gambiae *is estimated at several hundred thousand [43], which falls within the range of *Drosophila *estimates and might explain similarities in levels of genetic diversity in both species. We also observed a slightly reduced diversity in *A. arabiensis *compared to *A. gambiae *and in the M form of *A. gambiae *compared to the S form. Reduced genetic diversity in *A. arabiensis *was already observed previously [20,44], but colonization and further maintenance of *A. arabiensis *for several generations in an insectary might have added to this trend through increased genetic drift. Alternatively, long term effective population sizes differences might be expected between these species, in light of their distinct bionomics and distribution across Africa [45,46].

Patterns of genetic diversity can be influenced by differences in recombination rate across the genome, with a higher genetic diversity and a faster evolution rate expected in high recombination regions [47,48]. Our results must therefore be interpreted taking into account this potential heterogeneity along the *A. gambiae *genome. The known factors influencing recombination rate in *A. gambiae *are the chromosomal inversions and the proximity to centromeres and telomeres [44,49]. The distribution of immune related genes on the genome did not show any aggregation in particular regions (Figure 1), therefore we did not expect that the diversity pattern observed in immune related genes would be a consequence of heterogeneity of recombination rates along the genome. However, considering genes position on the genome sheds light on some evolutionary processes in *A. gambiae*. Paracentric chromosomal inversions are very abundant in the *A. gambiae *complex. More than 120 polymorphic inversions have been detected in natural populations [49,50]. Ten inversions are fixed in the different species of the complex and can be used to differentiate individual specimens. Several lines of evidence suggest that these chromosomal arrangements are incidental to ecotypic adaptation and may be directly involved in the past and current speciation processes occurring within this species complex, mainly because recombination suppression between alternative chromosomal arrangements might protect arrays of co-adapted genes [46,51-56]. Mathematical models for inversion-based local adaptation and/or speciation predict lower genetic diversity within species in the chromosomal region involved in speciation, and higher divergence between species [57,58]. In agreement with these models, we found evidence for reduced variability and higher genetic divergence between species on the X-chromosome. Indeed, *A. arabiensis *and *A. gambiae *are differentiated by a fixed chromosomal arrangement (Xag) that inverts a large part of the X chromosome [49]. Reduced effective population size for the X chromosome is not sufficient to explain the observed pattern which is therefore in agreement with previous studies demonstrating a "large × effect" on differentiation between these sibling species [44,59]. The "large × effect" hypothesis assumes the existence of speciation genes on the X chromosome responsible for ecological and/or behavioral adaptations that affect interspecific mating and/or hybrid fitness.

No X chromosome inversions, detectable at the cytogenetic level, distinguish the M and S forms of *A. gambiae*. Moreover, our samples were collected from an area of South Cameroon where *A. gambiae *M and S are known to be homosequential for the standard karyotype on all autosomes [60-62]. Accordingly, levels of genetic differentiation (single gene Fst estimates) were generally much lower between the M and S forms of *A. gambiae *than they were between these populations and *A. arabiensis*, and only occasionally did reach statistical significance. However, consistent with recent evidence for increased differentiation due to reduced recombination in the centromeric region of the X chromosome of *A. gambiae *[44,62-64], the only fixed SNP we observed between M and S maps to the proximal region of the X chromosome (X5D), a region that is considered as a "speciation island" between the M and S forms of *A. gambiae *[62]. Except for the X chromosome, we observed comparable genetic diversity across the entire genome. We did not detect any centromere or telomere effect, but a higher density of genetic markers would be necessary to draw firm conclusions about reduced diversity in these regions. The constant distribution of genetic diversity on autosomal chromosomes observed in the present study is in contrast with results of the *A. gambiae *genome project, where a highly variable distribution was observed [19]. The genome sequencing project utilized the PEST strain that was established from a mix of several natural populations of the M and S forms of *A. gambiae*, maintained under insectary conditions for several years and exposed to bottlenecks and selections [65]. It is likely that the uneven distribution of diversity in the PEST strain resulted from maintenance in the insectary, and that diversity in natural populations is more evenly distributed. It must be kept in mind, however that the present study focuses on populations from only one location for each species and might reflect only a portion of the natural genetic diversity of the species. Moreover, Cameroon might be an area where the M and S forms of *A. gambiae *have achieved one of the highest level of genetic differentiation observable throughout the species range, as was first described by Wondji et al. [61] using microsatellite data and further expanded by Turner et al.[62,66], using sequence data. Overall, our estimates of Fst between species/molecular forms are in strong agreement with those of Wondji et al. [61] and Turner et al. [62,66], who detected one of the highest and most significant level of genetic differentiation between the M and S forms of *A. gambiae *observable throughout the species range.

Tests for departure from neutrality are based on the assumption of mutation-migration-drift equilibrium. However, evidence for recent population expansion and radiation has been found in both *A. gambiae *and *A. arabiensis *[67,68]. Unstable demographic history can produce patterns of genetic variation indistinguishable from those of selection [69,70]. However, demographic history affects similarly the entire genome, while selection is locus-specific. For example, rapid population expansion is expected to result in highly negative Tajima D values, as a consequence of the rapid increase in number of polymorphic sites (S), and excess of low frequency alleles that have little effect on π [71]. This pattern was not observed in our dataset, where most computed Tajima D values were negative but not statistically significant. The Z test of selection and Ka/Ks ratios demonstrated a deficit of non synonymous mutations in most of the genes (immune related or not). The influence of population size changes on tests based on synonymous and non synonymous variations appears to be weak, even if not fully understood [35]. Therefore, it is most likely that the deficit of non synonymous mutations is due to generalized purifying selection acting on *A. gambiae *ORFs, probably reflecting functional constraints on the encoded proteins.

In *Drosophila*, several immunity genes revealed directional selection [22,72,73] and a broad comparison of immune system and non immunity genes supported the hypothesis that pathogens exert a selective pressure on the immune system [74]. Evidence for directional selection driving evolution of the immune system in *Drosophila *was consistent with the "arms race" model of co-evolution [75]. In this model, the pathogen constantly evolves to escape the host's immune response and, in turn, the hosts' immune system evolves to better control infections. Such dynamic iterative interactions would promote rapid evolution in the genes involved in pathogen-host interactions with rapid rise in frequency of selectively advantageous alleles and high turn-over between alleles leaving insufficient time for the accumulation of neutral polymorphism [76,77]. In contrast with data in *Drosophila*, our results did not detect a pattern of directional selection in innate immunity genes of *A. gambiae*. Although it is likely that different evolutionary forces are at play in these organisms, our inconclusive results probably reflect the limited statistical power of our dataset. Indeed, evidence for a higher rate of evolution in immune related genes compared to housekeeping genes in *Drosophila *was generated through a powerful sequence dataset and the results of the tests of selection was right below the 5% statistical significance thresholds [74]. In our data, the statistical power was limited by the small number of fixed mutations detected between species, pointing towards the necessity of using a more distant outgroup than *A. arabiensis *for evolutionary studies in *A. gambiae *[24]. Suitable outgroup species should diverge sufficiently to allow powerful selection tests without reaching mutation saturation. There is evidence that members of the *A. gambiae *complex are so closely related to each other that their low level of divergence would limit the statistical power of any conventional test of selection. Moreover, genetic introgression between well established species within the complex further overshadows their phylogenetic relationships and reduces divergence time [78]. In contrast, species of the Pyretophorus series other than *A. gambiae *complex members appeared too divergent to represent appropriate outgroups [24]. The identification of a suitable outgroup for comprehensive and powerful evolutionary studies in the *An. gambiae *complex is still pending but it is likely that ongoing whole-genome sequencing efforts and increased interest in this burgeoning field will soon provide appropriate candidate species and allow revision of previously inconclusive inferences.

In *A. gambiae *as well as in *Drosophila*, the level of genetic diversity appeared to be similar between immune related genes and control genes and between functional categories of genes involved in immunity. Balancing selection does not drive the evolution of the immune system (the specific case of TEP1 in *A. gambiae *is discussed below). This is contrasting with the pattern of selection observed in vertebrates, in which genes involved in defense mechanisms are under balancing selection in addition to directional selection. In vertebrates, the system of recognition of acquired immunity requires a large diversity of major histocompatibility complex genes to bind large diversity of antigens [79]. As such, the pattern of variability in *A. gambiae *and *Drosophila *immune systems is consistent with the recognition of relatively few motifs conserved across broad ranges of pathogens. However, the recent discovery of hypervariable immunoglobulin domain-encoding genes, Dscam, capable of producing pathogen-specific splice [80,81] opens new insights into recognition system in insects suggesting specific recognition of a spectrum of pathogens. Future investigations of molecular evolution will determine the selective forces at play on such genes and will help understanding their role in pathogen recognition.

One of the immune-related genes, TEP1, showed a unique pattern of variation. It displayed the highest level of genetic diversity among the genes we investigated and a significant positive value of the Tajima D statistic suggesting maintenance of divergent alleles. A previous study [7] revealed two highly differentiated TEP1 alleles that were initially mistaken as distinct genes, in the first version of the genome assembly. Crosses between laboratory strains showed Mendelian inheritance of these allelic forms, TEP1s and TEP1r, which are associated with two *A. gambiae *strains susceptible and refractory to *P. berghei*, respectively [7]; it was hypothesized that the alternative alleles are causally related to these phenotypes. The diversity we observed in TEP1 can be the result of balancing selection and would be reminiscent of selection for diversity in acquired immunity. However, gene conversion between different genes in the TEP family might result in similar patterns of diversity and further investigation is needed to disentangle these hypotheses. Nonetheless, our results emphasize the importance of the TEP1s and TEP1r alleles, and demonstrate these to segregate in natural populations. However, their role in determining the susceptibility of *A. gambiae *to *Plasmodium *infection remains to be established, as their segregation in laboratory strains could be due to increased genetic drift at the onset and throughout the colonization process.

## Conclusion

Through the sequencing of 109 fragments of genes in *A. gambiae*, we identified 3,214 SNPs that are relevant markers for future phenotype-association studies. The pattern of genetic variability showed little evidence for maintenance of protein variation by balancing selection in *A. gambiae *immune system. It revealed strong purifying selection as the main force driving evolution of the *A. gambiae *genome, probably as a result of functional constraints for protein integrity and activity. TEP1 showed a unique pattern of genetic diversity that could be the consequence of balancing selection or gene conversion.

## Methods

### Mosquito populations

*A. gambiae *s.s. larvae were collected in Simbock (03°51'N, 11°30'E), a South Cameroon village near Yaoundé, where both molecular forms M and S are sympatric [82]. The fact that the M and S populations were collected in a single village allows measuring genetic differentiation without bias due to geographical distance between collection sites. Larvae were reared in an insectary until adult emergence. *A. arabiensis *larvae were collected in Pitoa (09°24'N, 13°30'E), in North Cameroon [83] and the offspring were maintained in the insectary for approximately 10 generations, in 26–27 degrees Celsius, relative humidity 70–80% with 12 h/12 h light dark cycle. The number of *A. arabiensis *at each generation was always more than 100 individuals, avoiding strong bottleneck and genetic drift for the given number of generations. Anophelines were identified as members of the *A. gambiae *complex using morphological keys [45,84]. Species were identified using species-specific PCR [85] and the molecular forms of *A. gambiae *were distinguished by the PCR assay of Favia et al. [86]. Eight M molecular form females, 9 of the S molecular form and 8 *A. arabiensis *were used for sequence analysis.

### DNA/RNA isolation and sequencing

Coding regions are especially informative in evolutionary genetics and allow tests of selection based on comparison of synonymous (sSNPs) and non-synonymous (nsSNPs) mutations. Depending on the distribution of introns and exons, specific PCR assays for each gene were developed from coding regions of genomic DNA (gDNA) or complementary DNA (cDNA).

DNA was isolated from legs of adult females as described [21] and amplified with the Genomiphi kit (GE Healthcare, UK). This procedure conserves DNA polymorphism and does not alter SNP detection [87]. RNA was isolated from the same individuals (entire mosquitoes minus legs) by Trizol reagent (Invitrogen). After DNase I treatment, total RNA was reverse transcribed using the Superscript II kit (Life Technologies).

PCR assays were developed to amplify all or part of the coding regions of studied genes. Data on DNA sequence, genomic position and known polymorphism were obtained from the ENSEMBL website [88]. PCR primers were designed using Primer3 [89]. PCR reactions were performed in 50 μl solution containing 20 pmol of each primer, each dNTP at 0.2 mM, 2.5 mM MgCl_2_, 10 mM Tris-HCl (pH 8.3), 50 mM potassium chloride (KCl), 2 units of Taq polymerase and approximately 10 ng of template DNA. Amplification conditions included an initial 5 min 94°C denaturation, followed by 12 cycles at 94°C for 30 s, 65°C for 30 s, with a decrease of one degree per cycle, and finally 72°C for 1 min 30 s. They were followed by 25 cycles of 94°C for 30 s, 56°C for 30 s, and 72°C for 1 min 30 s. A final 72°C extension step lasted 10 min. The excess dNTPs were digested with Shrimp Alkaline Phopshatase and primers with ExonucleaseI (United State Biochemicals). Both strands were sequenced using the Bigdye terminator v3.1 cycle sequencing kit (Applied Biosystems) and an Applied Biosystems 3730 sequencer. Sequences were assembled and verified using SeqScape (Applied Biosystems).

### Data analysis

Sequence alignments were performed using the ClustalW included in MEGA 3.1 [90]. Non-coding regions were removed from analysis. Calculations were carried out after elimination of alignment gaps. Polymorphism analyses and molecular population genetic test statistics were calculated using DnaSP 4.10 [91] and MEGA 3.1.

For each population (hereafter *A. arabiensis*, *A. gambiae *M, *A. gambiae *S), we calculated the numbers of segregating sites (SS), informative segregating sites (ISS: polymorphisms found more than once in the dataset), and nsSNPs. Nucleotide diversity was estimated as the average pairwise nucleotide difference per site, considering all sites (π), synomymous sites only (πs) or non-synonymous sites only (πns) [92] and from the proportion of segregating sites θw [93]. Genetic diversity estimates were compared between groups of genes using the Mann-Whitney U test. Estimates derived from X-linked genes were adjusted for their lower effective population size by multiplying estimates by 4/3 because male mosquitoes carry only one copy of the X chromosome [e.g. [29]].

Divergence between species and genetic differentiation between *A. gambiae *molecular forms was assessed by sequence-based *F *statistics (Fst) analogous to Wright *F *statistics [94], calculated according to Hudson *et al *[95]. Levels of genetic divergence in groups of genes were compared using the Mann-Whitney U test. P values of average Fst across several loci were calculated by Fisher's method.

Molecular signatures of selection were searched using various statistical tests. The Tajima's D statistics [71] compares two estimators of genetic diversity, one based on the average number of differences between all pairs of sequences sampled (θ_π_) and the other based on the total number of polymorphic sites observed (θ_S_). If the population is at mutation-drift equilibrium and polymorphism is neutral, both estimators should be equal and the test statistic is zero. However, under selection or non-equilibrium, the two estimators will differ, and this difference reflects the mode of selection or the direction of change in population size. While θ_S _is only influenced by the number of segregating sites in the dataset, π is sensitive to allele frequencies at segregating sites, such that alleles at intermediate frequencies contribute more than alleles at low frequencies. Consequently, if a sample has an excess of rare variants (as a consequence of purifying selection or of population growth), θ_π _would be less than θ_S _and the statistic is negative. In contrast, if there is an excess of alleles at intermediate frequency (e.g. balancing selection or population bottleneck), Tajima's D statistic will be positive [70,71]. Comparison of the pattern observed across multiple independent genes allows distinguish locus-specific effects of selection from genome-wide patterns attributable to demographic changes. To avoid a possible bias due to mildly deleterious alleles towards low frequency variants, Tajima'D tests were computed using silent sites only.

To detect positive Darwinian selection (directional selection), we compared the number of synonymous substitutions per synonymous site (dS) and the number of non-synonymous substitutions per non-synonymous site (dN) [76] using bootstrapping in MEGA 3.1 (Z-test of selection). We took alternatively as the null hypothesis dS = dN (neutral hypothesis), dS<dN (positive selection) and dS>dN (purifying selection).

The Ka/Ks ratio compares the number of replacement substitutions per site (nsSNPs) and silent substitutions per site (sSNPs) among different populations [96,97]. This ratio is higher for genes under selection for beneficial amino acid changes. Ka/Ks ratios were calculated for each gene and for each pair of populations.

Under neutral evolution, the ratio of replacement to silent mutations that are fixed between species should equal the ratio of replacement to silent polymorphisms within species [98]. The MacDonald-Kreitman test uses a 2 × 2 contingency table to test differences in these ratios. This test could not be performed with each gene separately because, in most cases, the number of fixed polymorphisms was too low for contingency table computation. Therefore, the test was performed with the sum of fixed/polymorphic sSNPs and nsSNPs across all genes for each pair of populations, by using the 2 × 2 test of independence in DnaSP. Summing mutations across genes can lead to spurious cases of positive selection [34] the results must therefore be carefully interpreted

## Authors' contributions

AC contributed to the design of the study, carried out the experiments, analyzed the data and wrote the manuscript. SK and MM, participated in the sequencing. FS and IM helped in analysis and to draft the manuscript. AK participated in the sequence alignments. DF and FCK conceived of the study, participated in its design and coordination and helped to draft the manuscript. All authors read and approved the final manuscript.

## Supplementary Material

Additional file 1 **Characteristics of locus amplified for SNP detection in coding regions of immune related genes**. *: 1: Recognition, 2: Modulation, 3: Signal transduction, 4: Effector molecules according to Christophides *et al *[5] Click here for file

Additional file 2 Characteristics of locus amplified for SNP detection in coding regions of non immune related genes. Click here for file

Additional file 3 **Nucleotide polymorphism in immune related genes**. SS: number of segregating sites. ISS: number of informative sites (polymorphisms found more than once in the dataset). nsSNP: number of non synonymous SNPs. π: nucleotide diversity considering all sites. πs: nucleotide diversity considering synomymous sites only. πns: nucleotide diversity considering non-synomymous sites only. θw: the proportion of segregating site. Tajima's D test: D value, significant values are in bold characters if P < 0.05, and bold and underlined characters if P < 0.01. Z test of selection: test for purifying selection (Nei and Gojobori method)._: data not available due to a failure of sequencing or lack of polymorphism. Click here for file

Additional file 4 **Nucleotide polymorphism in non immune related genes**. SS: number of segregating sites. ISS: number of informative sites (polymorphisms found more than once in the dataset). nsSNP: number of non synonymous SNPs. π: nucleotide diversity considering all sites. πs: nucleotide diversity considering synomymous sites only. πns: nucleotide diversity considering non-synomymous sites only. θw: the proportion of segregating site. Tajima's D test: D value, significant values are in bold characters if P < 0.05, and bold and underlined characters if P < 0.01. Z test of selection: test for purifying selection (Nei and Gojobori method)._: data not available due to a failure of sequencing or lack of polymorphism. Click here for file

Additional file 5 **Divergence between populations detected on immune related genes**. Polymorphic sites: number of polymorphic sites within populations. Fixed sites: number of fixed divergence between populations. _: data not available due to a failure of sequencing. Click here for file

Additional file 6 **Divergence between populations detected on non immune related genes**. Polymorphic sites: number of polymorphic sites within populations. Fixed sites: number of fixed divergence between populations. _: data not available due to a failure of sequencing. Click here for file
